# Dose-Dependent Effects of L-Serine Supplementation on Boar Sperm Quality During Chilled and Cryopreserved Storage

**DOI:** 10.3390/ani15182670

**Published:** 2025-09-12

**Authors:** Vibuntita Chankitisakul, Himalai Saiyamanon, Wuttigrai Boonkum, Eakapol Wangkahart, Ruthaiporn Ratchamak

**Affiliations:** 1 Department of Animal Science, Faculty of Agriculture, Khon Kaen University, Khon Kaen 40002, Thailand; vibuch@kku.ac.th (V.C.); himalai@kkumail.com (H.S.); wuttbo@kku.ac.th (W.B.); 2 Network Center for Animal Breeding and Omics Research, Khon Kaen University, Khon Kaen 40002, Thailand; 3 Laboratory of Fish Immunology and Nutrigenomics, Applied Animal and Aquatic Sciences Research Unit, Division of Fisheries, Faculty of Technology, Mahasarakham University, Khamriang Sub-District, Kantarawichai 44150, Maha Sarakham, Thailand; eakapol.w@msu.ac.th; 4 Major of Animal Science, Department of Agricultural Technology, Faculty of Technology, Mahasarakham University, Kantarawichai 44150, Maha Sarakham, Thailand

**Keywords:** antioxidant, boar semen, L-serine, cryopreservation, cooled semen

## Abstract

Boar sperm are highly sensitive to oxidative and cold-induced stress during storage, which compromises their functional integrity. This study evaluated the dose-dependent effects of L-serine, an amino acid involved in glutathione synthesis and membrane stabilization, on boar sperm quality under chilled (17 °C) and cryopreserved conditions. Ejaculates from fertile boars were extended with Modena-based media supplemented with 0, 1, 3, 5, and 7 mM L-serine. Sperm quality parameters, including motility, viability, mitochondrial activity, acrosome integrity, lipid peroxidation (measured by malondialdehyde (MDA)), and antioxidant enzyme activity, were assessed over five days for chilled semen and post-thaw for cryopreserved samples. L-serine at 3 mM was most effective in maintaining chilled semen quality, as evidenced by superior motility, viability, and acrosome integrity by Day 5, accompanied by lower MDA levels and increased glutathione peroxidase and catalase activities. In contrast, 1 mM was optimal for cryopreserved semen, improving post-thaw motility, mitochondrial activity, and antioxidant defense while reducing lipid peroxidation. Higher concentrations (≥5 mM) consistently impaired sperm function, likely due to osmotic stress or redox imbalance. These findings indicate that L-serine enhances boar sperm preservation in a concentration- and method-specific manner, with 3 mM supplementation most effective for chilled storage, and 1 mM for cryopreservation. This study supports the inclusion of amino acid antioxidants in extender formulations to improve semen preservation in swine artificial insemination systems.

## Simple Summary

Maintaining the quality of boar semen during storage is crucial for effective breeding programs. However, current storage methods, especially cooling and freezing, can damage sperm cells due to temperature stress and harmful molecules, such as reactive oxygen species. This study explored whether L-serine supplementation could protect boar sperm during chilled and cryopreserved storage. We tested different concentrations and found that moderate supplementation (3 mM) best preserved sperm quality during chilling, while a lower dose (1 mM) showed the most favorable in vitro outcomes for cryopreservation. Excessive doses impaired sperm function. These results highlight the importance of dose adjustment for improving semen storage reliability in swine breeding programs. It is also noteworthy that chilled and cryopreserved storage induce sperm damage through distinct mechanisms, which may explain why the optimal concentration of L-serine differs between the two preservation methods.

## 1. Introduction

Efficient semen preservation is vital for swine artificial insemination (AI) systems, enabling rapid genetic dissemination and reproductive efficiency. However, boar spermatozoa are particularly vulnerable to cold and oxidative stress, limiting their viability during both chilled and cryopreserved storage [1,2]. This sensitivity stems mainly from their plasma membrane composition, which contains high levels of polyunsaturated fatty acids (PUFAs) and a low cholesterol-to-phospholipid ratio, predisposing sperm to lipid peroxidation, membrane destabilization, and motility loss under storage stress [3,4].

Oxidative stress, induced by reactive oxygen species (ROS), is a key contributor to sperm deterioration. Elevated ROS levels impair membrane integrity, reduce mitochondrial function, and compromise DNA, leading to diminished fertilizing ability [5]. To counteract this, antioxidant supplementation in semen extenders has emerged as a promising strategy. Several compounds, including glutathione, L-carnitine, and resveratrol, have shown potential to mitigate oxidative damage, though their efficacy is highly context- and dose-dependent [6,7].

L-serine is a conditionally non-essential amino acid involved in antioxidant defense and membrane biosynthesis. It contributes to glutathione production and the formation of structural phospholipids and sphingolipids [8,9,10]. Prior studies in poultry have shown that L-serine supplementation enhances post-thaw sperm quality [11,12], but research on its role in boar semen preservation remains limited. Moreover, boar sperm undergoes storage-induced stress that is highly dependent on temperature [13,14,15,16]. L-serine has both osmotic and metabolic functions. Its effects may vary under different thermal conditions, as temperature influences membrane fluidity, cell permeability, and redox balance. During chilling, L-serine may stabilize membranes and support mild antioxidant demand, whereas under cryogenic stress, excessive supplementation may exacerbate osmotic or oxidative injury [9,17].

Additionally, many previous studies on antioxidant use in boar semen have focused on a single concentration or preservation method, often lacking systematic comparisons across doses and storage types. Few have examined compounds like L-serine that simultaneously influence redox status and osmotic balance, further emphasizing the novelty of this investigation.

Despite its potential, the effects of L-serine on boar sperm quality across different preservation conditions have not been systematically investigated. Therefore, the objective of this study was to determine the dose-dependent effects of L-serine on sperm quality in boar semen subjected to either chilled or cryopreserved storage. We hypothesized that L-serine supplementation would enhance sperm viability and antioxidant defense in a concentration-specific manner, with the most effective concentrations for each storage method. Our findings aim to improve extender formulations and inform precision antioxidant strategies in swine reproduction.

## 2. Materials and Methods

All experimental procedures and animal handling protocols were conducted in accordance with ethical guidelines and were approved by the Animal Ethics Committee of Khon Kaen University, Thailand (Approval No. IACUC-KKU 49/68).

### 2.1. Animals and Management

Semen was collected from three fertile boars (Duroc, Landrace, and Large White), aged 1–2 years. The boars were housed individually under standardized management conditions in an open-housing system with natural ventilation. Each boar had ad libitum access to clean drinking water via an automated watering system and was fed a balanced diet formulated to meet the nutritional requirements for optimal semen production. Body condition scores ranged between 3.0 and 3.5 on a standardized 1-to-5 scale.

### 2.2. Semen Extender

A Modena extender was used as the base formulation for both chilled and cryopreserved semen preservation in this study. It served as the primary diluent for initial semen dilution, sperm concentration adjustment, and antioxidant supplementation.

The Modena extender is composed of the following constituents: 2.750 g D-glucose, 0.690 g Tri-sodium citrate, 0.100 g sodium hydrogen carbonate, 0.235 g EDTA disodium salt, 0.565 g Tris (Methylamine), 0.290 g citric acid, 0.060 g penicillin and 0.100 g streptomycin.

### 2.3. Semen Collection and Preparation

Ejaculates were collected as bulk samples from each boar (n = 3) twice weekly using the standard gloved-hand technique [16]. Across the experimental period, a total of 21 ejaculates (7 ejaculates per boar) were obtained. Semen volume, sperm concentration, and progressive motility were immediately evaluated using standard laboratory protocols. Only ejaculates exhibiting ≥70% progressive motility were selected for experimental use.

### 2.4. Fresh Semen Evaluation

Semen volume was determined immediately after collection by measuring the sperm-rich fraction in a sterile 50 mL graduated centrifuge tube. The volume of each ejaculate was recorded in milliliters (mL) [18].

Sperm concentration was measured using the SpermaCue^®^ photometer (Minitube of America, Inc., Verona, WI, USA). A 30 μL aliquot of semen was loaded into the sample well of a disposable cuvette. The cuvette was then inserted into the photometer via the slider mechanism. The sperm concentration, displayed in millions of sperm per milliliter (×10^6^ sperm/mL), was automatically calculated and recorded.

Progressive motility was assessed by placing a 5–10 μL drop of semen on a clean glass slide without a coverslip and examining under a compound microscope at 10× magnification (Olympus CH30, Tokyo, Japan). Progressive motility was defined as the percentage of sperm moving actively in a forward linear direction.

### 2.5. Experimental Design

To determine the effective concentrations of L-serine for boar semen preservation under both chilled storage and cryopreservation conditions, ejaculates that met the minimum quality criteria during initial evaluation were selected. Each qualified ejaculate was divided into two aliquots: one for chilled storage at 17 °C, and the other for cryopreservation in liquid nitrogen (LN_2_).

For chilled storage, semen samples were extended using the Modena extender supplemented with L-serine at final concentrations of 0 (control), 1 mM, 3 mM, 5 mM, and 7 mM. The final sperm concentration in all treatments was adjusted to 3 × 10^7^ sperm/mL. Samples were stored at 17 °C, and aliquots from each treatment were assessed on Days 1, 3, and 5 of storage.

For cryopreservation, semen samples were diluted with Freezing Extender I containing the same five L-serine concentrations: 0 mM (control), 1 mM, 3 mM, 5 mM, and 7 mM. The post-thawed samples were subsequently assessed.

In both preservation methods, the following parameters were evaluated: progressive sperm motility, viability, acrosome integrity, mitochondrial membrane potential, lipid peroxidation (MDA concentration), and antioxidant enzyme activities. Each treatment was replicated seven times using semen from 21 ejaculates (n = 21).

### 2.6. Semen Cooling Storage

The cooling storage procedure used in this study was as described by Vongpralub et al. [19]. After the semen was diluted with the Modena extender to reach the final sperm concentration, the aliquots were dispensed into sterile 5 mL tubes and stored at 17 °C in a temperature-controlled refrigerator. The storage temperature was continuously monitored using a calibrated digital thermometer to ensure consistent thermal conditions throughout the experiment.

### 2.7. Semen Cryopreservation

The freezing procedure used in this study was as described by Ratchamak et al. [20]. Briefly, semen was initially diluted with a Modena extender at a 1:1 (*v*/*v*) ratio and gradually cooled to 15 °C over a period of 120 min. The cooled semen was then centrifuged at 800× *g* for 10 min at 15 °C to separate sperm cells from the seminal plasma. The resulting sperm pellet was resuspended in Freezing Extender I (1:2, *v*/*v*), composed of 80 mL of 11% lactose solution and 20 mL of egg yolk, to achieve a final sperm concentration of 1.5 × 10^9^ sperm/mL. This suspension was further cooled to 5 °C over 90 min.

Subsequently, the samples were mixed with Freezing Extender II (2:1, *v*/*v*), which consisted of 89.5% Freezing Extender I, 9% glycerol, and 1.5% Equex STM paste, to achieve a final concentration of 1 × 10^9^ sperm/mL. The semen was loaded into 0.5 mL plastic straws, which were horizontally positioned on a freezing rack 11 cm and 5 cm above the surface of LN_2_, corresponding to temperatures of approximately −35 °C and −120 °C, respectively. The straws were maintained at these levels for 5 min and 10 min, respectively, before being plunged into LN_2_ for cryogenic storage.

For the post-thaw analysis, the semen straws were thawed in a 5 °C water bath for 5 min. Thawed samples were then diluted 1:4 (*v*/*v*) in chilled Modena™ extender (5 °C) and incubated in a 37 °C water bath for 30 min prior to the sperm quality evaluation.

### 2.8. Post-Thaw Semen Evaluation

#### 2.8.1. Sperm Motility

Progressive sperm motility was evaluated using a Computer-Assisted Semen Analyzer (CASA; Hamilton Thorne Biosciences, version 12 TOX VIOS) with the following settings: frames per second, 60 Hz; number of frames, 30; head brightness min, 170; minimum cell size, 5 μm^2^; and maximum cell size, 50 μm^2^. Sperm with an average path velocity (VAP) less than 20 μm/s were classified as non-motile. For each evaluation, 3–5 μL of semen was loaded into a pre-warmed (37 °C) counting chamber, and at least 300 spermatozoa from five fields were examined. Progressive motility was defined as sperm moving actively in a forward linear trajectory [21].

#### 2.8.2. Sperm Viability, Mitochondrial Function, and Acrosome Integrity

Sperm viability, mitochondrial membrane potential, and acrosome integrity were assessed using a triple fluorescent staining as described by Ratchamak et al. [21]. Propidium iodide (PI), JC-1 (5,5′,6,6′-tetrachloro-1,1′,3,3′-tetraethylbenzimidazolylcarbocyanine iodide), and FITC-PNA (fluorescein isothiocyanate-conjugated peanut agglutinin) were used to evaluate viability, mitochondrial function, and acrosome status, respectively.

Briefly, 300 μL of semen was sequentially incubated with 2.5 μL PI (5 min, 37 °C), 2 μL JC-1 (10 min, 37 °C), and 2 μL FITC-PNA (15 min, 37 °C, dark). A 3–5 μL aliquot was mounted on a slide with a coverslip, and 300 spermatozoa were evaluated immediately under a fluorescence microscope (Olympus IX71, Tokyo, Japan) at 400× magnification [16]. PI staining was used to assess sperm viability. Non-viable spermatozoa with compromised plasma membranes fluoresced red in the head region, whereas viable spermatozoa with intact membranes remained unstained. Acrosome integrity was evaluated using FITC-PNA, with spermatozoa displaying yellow-green fluorescence in the acrosomal region classified as acrosome-damaged. Mitochondrial membrane potential was assessed using JC-1 staining, where spermatozoa exhibiting red-orange fluorescence in the midpiece were considered to have high mitochondrial membrane potential, indicating functional mitochondrial activity.

#### 2.8.3. Lipid Peroxidation

Lipid peroxidation was quantified as malondialdehyde (MDA) concentrations following Ratchamak et al. [21]. In brief, 250 µL of semen was incubated with 250 µL each of 0.25 mM ferrous sulfate and 0.25 mM ascorbic acid at 37 °C for 1 h. After adding 1 mL of 15% trichloroacetic acid and 1 mL of 0.375% thiobarbituric acid, samples were boiled for 10 min, cooled, and centrifuged at 800× *g* for 10 min. MDA in the supernatant was measured spectrophotometrically at 532 nm (Specord 250 plus; Analytik Jena, Jena, Germany) and expressed as μmol/mL.

#### 2.8.4. Antioxidant Enzyme Activity

To determine the enzyme activities, semen was centrifuged at 4000× *g* for 10 min at room temperature to separate seminal plasma, which was stored at −20 °C until analysis. Enzyme activities were measured in 96-well plates using a microplate reader (VersaMax™, Molecular Devices), and expressed in units per milliliter (U/mL) unless otherwise stated.

##### Total Antioxidant Capacity (T-AOC)

T-AOC was assessed via the 2,2-diphenyl-1-picrylhydrazyl (DPPH) radical scavenging assay as described by Karirat et al. [22]. Briefly, 20 μL of seminal plasma was mixed with 180 μL of 0.2 mM DPPH ethanolic solution and incubated at 30 °C for 30 min in the dark. Absorbance was measured at 520 nm using a microplate reader (iMark™, Bio-Rad). Dextran was used as the standard. T-AOC (%) was calculated as: T-AOC (%) = [1 − (OD_sample_ − OD_blank_)/OD_control_] × 100, where OD = optical density, OD_blank_ contained deionized water, OD_control_ contained all reagents without the sample, and OD_sample_ contained all reagents with the seminal plasma sample.

##### Glutathione Peroxidase (GPx)

GPx activity was measured using a coupled enzyme assay [23]. Seminal plasma (20 μL) was mixed with 20 μL of 0.1% Triton X-100 in Phosphate-Buffered Saline (PBS), pH 7.4), 20 μL of solution containing 24 μM reduced glutathione (GSH), 4.8 μM nicotinamide adenine dinucleotide phosphate (NADPH), and 12 U glutathione reductase, followed by 35 μL of 1% H_2_O_2_. After incubation at room temperature for 3 min, absorbance at 450 nm was recorded at 30 and 90 s. GPx activity (U/mL) was calculated as: OD_450_/0.00622.

##### Superoxide Dismutase (SOD)

SOD activity was determined by the epinephrine auto-oxidation method [24]. A 20 μL aliquot of seminal plasma was added to 200 μL of reaction mixture (sodium carbonate buffer, pH 10.2, containing 30 mM epinephrine dissolved in HCl). Absorbance was recorded at 490 nm at 30 and 90 s. SOD activity (U/mL) = [100 − (ΔOD_sample_/ΔOD_control_ × 100)] × 3.75, where ΔOD = change in optical density; ΔOD_sample_ = change in absorbance of the seminal plasma sample; ΔOD_control_ = change in absorbance of the control.

##### Catalase (CAT)

CAT activity was measured as described by Weydert and Cullen [25]. A 10 μL aliquot of seminal plasma was mixed with 50 μL of 10% H_2_O_2_ and 50 μL of PBS (pH 7.4). Absorbance was measured at 240 nm at 20 and 80 s. CAT activity (U/mL) was calculated as OD_1_ − OD_2_/0.0008, where OD_1_ = absorbance at 20 s; OD_2_ = absorbance at 80 s.

##### Glutathione S Transferase (GST)

GST activity was assessed according to Zablotowicz et al. [26]. Seminal plasma (25 μL) was combined with 100 μL of 100 mM GSH in PBS (pH 7.4), incubated at 30 °C for 2 min, then mixed with 25 μL of 100 mM CDNB (1-chloro-2,4-dinitrobenzene). Absorbance was measured at 340 nm. The formula used to calculate the GST activity was as follows: GST activity (U/mL) = (OD_340_/0.006)/4.

##### Glutathione Reductase Activity (GRD)

GRD activity was measured following Weydert and Cullen [25]. Fifty μL of seminal plasma was mixed with 70 μL of reaction mixture (dH_2_O, TE buffer, and 18 mM oxidized glutathione, GSSG), incubated at room temperature for 2 min, then 50 μL of 3 mM NADPH was added to initiate the reaction. Absorbance was measured at 450 nm, and GRD activity (U/mL) was calculated as OD_450_/0.00833.

### 2.9. Statistical Analysis

Data for semen quality traits (progressive motility, viability, acrosome integrity, mitochondrial membrane potential, lipid peroxidation, and antioxidant enzyme activities) were analyzed using a randomized complete block design, with sires as blocks. Analysis of variance was performed, and treatment means were compared using Tukey’s multiple range test. Normality of residuals was evaluated with the UNIVARIATE procedure (NORMAL option), and homogeneity of variances was tested using Levene’s test. All analyses were conducted using SAS software (Version 9.0, SAS Institute Inc., Cary, NC, USA). Differences among treatments were considered statistically significant at *p* < 0.05.

## 3. Results

### 3.1. Fresh Semen Quality of Boars

The initial evaluation of ejaculates confirmed that all semen samples were within the normal physiological range for boar semen quality. The mean ejaculate volume was 200.47 ± 11.93 mL, with an average sperm concentration of 344.97 ± 21.98 × 10^6^ sperm/mL. Progressive motility averaged 81.42 ± 2.41%. All samples exceeded the minimum threshold of 70% progressive motility and were therefore considered suitable for inclusion in the experimental trials.

### 3.2. Effect of L-Serine on Semen Quality During Chilled Storage

As shown in Table 1, L-serine supplementation exerted a significant, concentration-dependent effect on boar semen quality during chilled storage at 17 °C for up to 5 days. Semen was divided into a control group (0 mM) and four treatment groups (1, 3, 5, and 7 mM L-serine). Among all concentrations, 3 mM consistently preserved sperm function better than the control and other treatments (*p* < 0.05), maintaining the highest levels of progressive motility, viability, mitochondrial activity, and acrosome integrity across all time points.

On Day 1, sperm quality parameters in all L-serine groups were comparable with the control (*p* > 0.05) or showed slight improvement. As storage time increased, differences among groups became apparent: by Days 3 and 5, the 3 mM group exhibited significantly higher functional parameters than the control, 1 mM, 5 mM, and 7 mM groups (*p* < 0.05). By Day 5, sperm quality was best preserved in the 3 mM group, whereas the 5 mM and 7 mM groups showed significant declines compared with both the control and 3 mM groups (*p* < 0.05), indicating detrimental effects at excessive concentrations.

Overall, higher concentrations (5 and 7 mM) led to progressive deterioration in all quality parameters compared with 3 mM (*p* < 0.05), possibly due to osmotic imbalance or redox stress. This pattern supports a biphasic response in which moderate supplementation (3 mM) is protective, whereas higher concentrations are harmful.

### 3.3. Lipid Peroxidation and Antioxidant Enzyme Activities During Chilled Storage

As shown in Table 2, lipid peroxidation, assessed by MDA levels, increased progressively during storage in all groups. However, this increase was significantly attenuated in the 3 mM group, which consistently showed the lowest MDA concentrations across all time points (*p* < 0.05), indicating a strong protective effect of 3 mM L-serine against oxidative lipid damage.

The activities of key antioxidant enzymes also varied among concentrations. The 3 mM group maintained the highest GPx and CAT activities throughout storage (*p* < 0.05), supporting its role in enhancing redox defense. By contrast, SOD activity was markedly increased in the 7 mM group (*p* < 0.05), but this was not accompanied by similar increases in GPx or CAT, suggesting an imbalance in the antioxidant response at excessive concentrations.

In contrast, GST and GRD activities did not differ significantly among groups or across storage days (*p* > 0.05), suggesting that these enzymes were not responsive to L-serine supplementation under chilled conditions.

### 3.4. Effect of L-Serine on Sperm Quality After Cryopreservation

**Figure 1:**
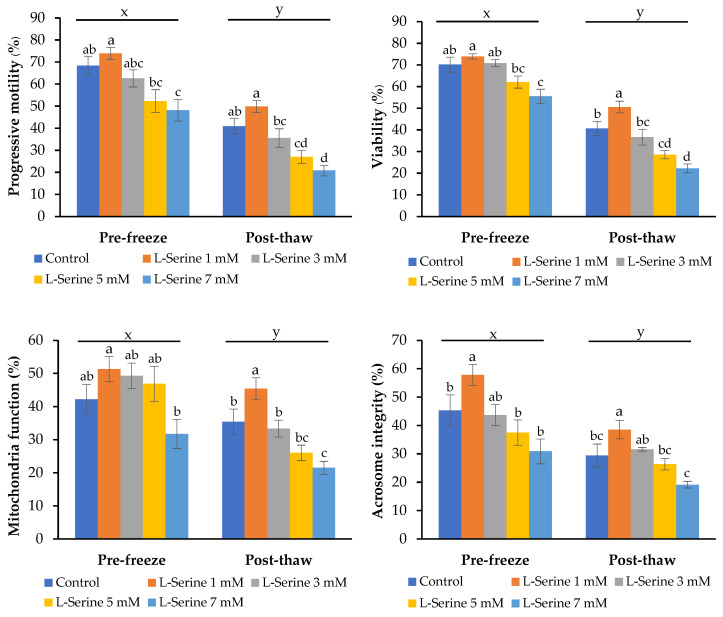
Effects of L-serine supplementation on sperm quality before freezing (pre-freeze) and after thawing (post-thaw) (values with different superscripts (x, y) indicate significant differences between pre-freeze and post-thaw values in the same treatment group (*p* < 0.05). Values with different lowercase superscript (a–d) indicate significant differences between L-serine levels within the same stage (*p* < 0.05).

As shown in Figure 1, cryopreservation significantly reduced sperm quality in all groups compared with pre-freeze values (*p* < 0.05). However, supplementation with 1 mM L-serine markedly improved post-thaw sperm function compared with the control group (*p* < 0.05), maintaining higher levels of progressive motility, viability, mitochondrial activity, and acrosome integrity.

In contrast, 3 mM L-serine did not improve post-thaw quality compared with the control (*p* > 0.05), whereas the 5 and 7 mM groups showed significantly lower sperm function than both the control and 1 mM groups (*p* < 0.05), indicating detrimental effects at excessive concentrations.

### 3.5. Lipid Peroxidation and Antioxidant Enzyme Activities After Cryopreservation

**Figure 2:**
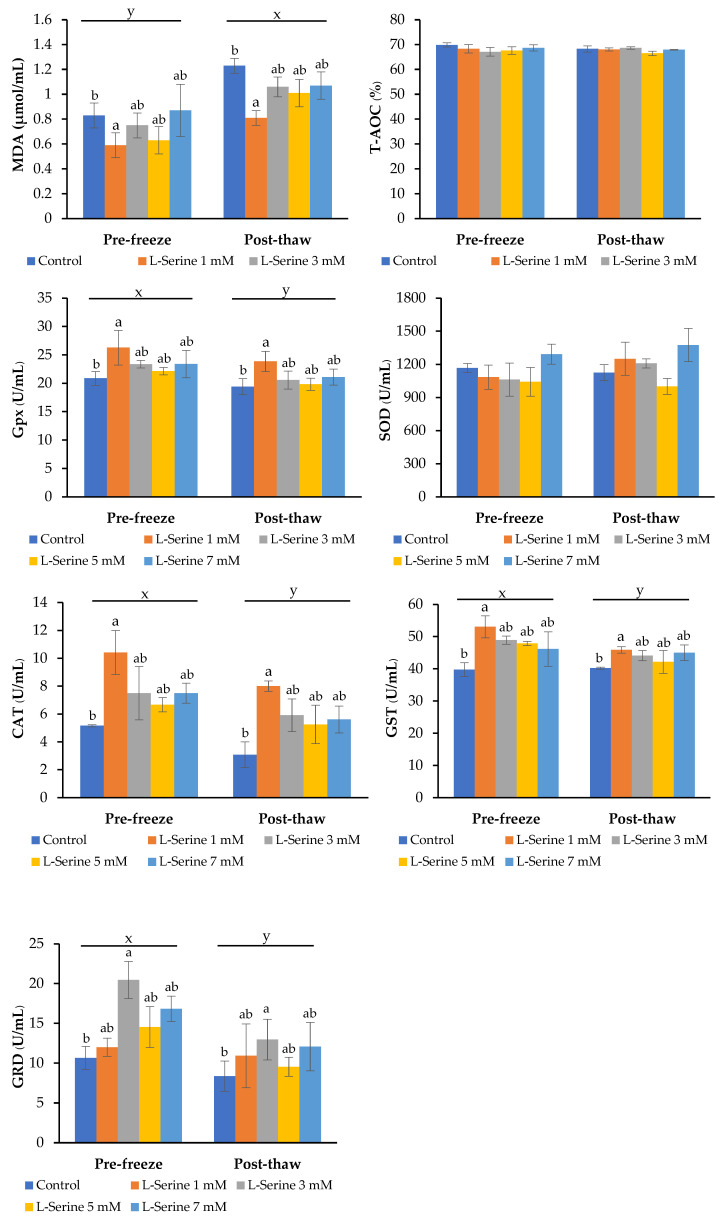
Effects of L-serine supplementation on lipid peroxidation and antioxidant enzyme activities in boar semen before freezing and after thawing. Values with different lowercase superscript (x, y) indicate significant differences between the pre-freeze and post-thaw stages (*p* < 0.05). Values with different lowercase superscript (a, b) indicate significant differences between the L-serine concentrations within the same stage (*p* < 0.05).

As shown in Figure 2, post-thaw MDA concentrations increased significantly in all groups compared with pre-freeze values (*p* < 0.05). However, the 1 mM L-serine group exhibited the lowest MDA levels after thawing (*p* < 0.05), indicating superior protection against lipid peroxidation compared with the control and higher concentrations (3, 5, and 7 mM).

Antioxidant enzyme activities also varied among treatments. The 1 mM group maintained significantly higher GPx, CAT, and GST activities post-thaw compared with the control and other concentrations (*p* < 0.05), reflecting enhanced redox buffering capacity. In contrast, SOD activity was markedly increased in the 7 mM group (*p* < 0.05), but this was not accompanied by improvements in sperm quality, suggesting a stress-induced compensatory response rather than a functional benefit.

GRD activity did not differ significantly among the groups or time points (*p* > 0.05), indicating that this enzyme was not responsive to L-serine supplementation under cryopreservation conditions.

Overall, these findings confirm that 1 mM L-serine was the most effective concentration for maintaining antioxidant balance and protecting against oxidative damage during cryopreservation, while higher concentrations may trigger stress responses without improving sperm quality.

## 4. Discussion

### 4.1. Dose-Dependent Effects of L-Serine on Boar Semen Preservation

Boar semen preservation, whether by chilled storage or cryopreservation, induces oxidative stress through distinct mechanisms. Chilled storage entails prolonged exposure to sub-physiological temperatures, during which residual mitochondrial activity generates low but continuous levels of ROS [27]. By contrast, cryopreservation causes acute oxidative and mechanical damage during freezing and thawing [28].

The present study demonstrated that L-serine supplementation significantly enhanced boar sperm quality under both storage conditions, but in a concentration- and method-specific manner. The protective effects were most pronounced at 3 mM during chilled storage and at 1 mM under cryopreserved conditions, which preserved key functional parameters—motility, viability, mitochondrial function, and acrosome integrity—better than control or higher-dose treatments in this study.

Notably, supplementation at ≥5 mM consistently impaired sperm quality across both preservation methods. This biphasic response suggests that L-serine exerts beneficial antioxidant effects at low-to-moderate concentrations but induces osmotic or redox imbalance when supplied in excess.

### 4.2. Antioxidant Mechanisms of L-Serine

The beneficial effects of L-serine supplementation on boar sperm quality can be attributed to several interconnected mechanisms that mitigate oxidative stress.

First, L-serine serves as a biosynthetic precursor for glycine and cysteine, both of which are required for glutathione (GSH) synthesis [8,10,29]. GSH represents the major non-enzymatic antioxidant defense in spermatozoa, directly neutralizing ROS and acting as a cofactor for GPx [8,29,30,31]. In the present study, effective L-serine supplementation was associated with increased GPx and GST activities, consistent with improved detoxification of lipid peroxides.

Second, L-serine contributes to NADPH generation via the folate-mediated one-carbon cycle, thereby supporting the regeneration of reduced GSH and thioredoxin [10,32,33]. This enhances the activities of enzymatic antioxidants such as CAT, GPx, and GRD. Indeed, L-serine-treated groups exhibited elevated activities of these enzymes together with reduced MDA levels. Interestingly, however, exaggerated SOD activity at the highest dose (7 mM), without corresponding increases in GPx or CAT, may have led to hydrogen peroxide accumulation, thereby explaining the decline in sperm quality observed at excessive supplementation.

Third, L-serine is a substrate for phospholipids and sphingolipids, which are critical for maintaining sperm plasma membrane stability and acrosome integrity during storage [34]. These membrane-stabilizing effects may act synergistically with antioxidant defenses to preserve sperm functional capacity.

Finally, mitochondrial function—which provides ATP to sustain motility—appeared better preserved in L-serine-supplemented semen, as indicated by higher mitochondrial membrane potential (MMP). Such protection may result from the combined antioxidative and membrane-stabilizing actions of L-serine [29,30]. Comparable findings were reported by Hu et al. [35], who showed that protocatechuic acid improved mitochondrial function and antioxidant enzyme activity in boar sperm via the AMPK/PGC-1/Nrf1 signaling pathway. Although our study did not directly evaluate molecular signaling, the preservation of MMP observed here suggests that L-serine may exert similar protective effects on sperm mitochondrial physiology.

Despite these interpretations, it should be noted that intracellular glutathione concentrations, phospholipid composition, and osmotic pressure were not measured directly in the present study. Instead, proxy indicators were employed to support these conclusions. GPx and GRD activities provide indirect evidence of glutathione utilization and regeneration, while acrosome integrity, plasma membrane integrity, mitochondrial activity, and MDA levels are reliable markers of membrane stability and oxidative resilience. Moreover, the potential osmotic role of L-serine is supported by previous reports describing amino acid-derived osmolytes as contributors to cryoprotection [36].

Taken together, these enzymatic and non-enzymatic mechanisms likely underpin the observed improvements in sperm motility, viability, and acrosome integrity during both chilled and cryopreserved storage.

### 4.3. Chilled Semen: Short-Term Preservation Benefits

During chilled storage at 17 °C, boar sperm are progressive exposed to oxidative stress [37,38]. In this study, supplementation with 3 mM L-serine effectively mitigated these oxidative effects during the early stages of storage, as evidenced by lower lipid peroxidation levels and enhanced sperm function compared with the control and higher-dose groups.

MDA concentrations, a key marker of oxidative lipid damage, remained significantly lower in the 3 mM group for the first three days but increased by Day 5, suggesting a time-limited efficacy as antioxidant reserves became depleted. Functional parameters showed a similar pattern, with superior motility, viability, mitochondrial activity, and acrosome integrity maintained up to 72 h, followed by a decline thereafter.

Enzymatic analyses further supported these findings: GPx and CAT activities were significantly elevated in the 3 mM group, indicating enhanced neutralization of peroxides. However, despite comparable increases in GPx and CAT at 5 mM, no functional benefit was observed, implying that excessive supplementation disrupted redox balance or imposed osmotic stress. A particularly striking observation was the markedly elevated SOD activity in the 7 mM group (up to 1500 U/mL). In the absence of proportional increases in GPx or CAT, this imbalance likely promoted hydrogen peroxide accumulation [39,40,41], helping to explain the decline in sperm quality at higher concentrations.

Overall, these results indicate that 3 mM L-serine provides optimal antioxidant protection during short-term chilled storage. In contrast, supra-physiological concentrations (≥5 mM) induce enzymatic and osmotic disturbances that compromise sperm integrity.

### 4.4. Cryopreserved Semen: Antioxidant Enzyme Responses to Cryopreservation

In this study, L-serine supplementation demonstrated a clear dose-dependent effect on post-thaw sperm quality. Among the tested concentrations, 1 mM L-serine was the most effective in preserving motility, viability, mitochondrial activity, and acrosome integrity better than the control and higher-dose groups.

Biochemical assessments reinforced this protective role. The MDA levels were significantly reduced in the 1 mM group both before freezing and after thawing, indicating lower lipid peroxidation. Concurrently, antioxidant enzyme activities (e.g., GPx, CAT) were maintained at higher levels, suggesting more efficient detoxification of ROS during cryogenic stress. These findings are consistent with previous studies highlighting the importance of sustaining glutathione and thiol redox pathways during sperm cryopreservation [42,43].

Nonetheless, a consistent decline in antioxidant enzyme activity post-thaw was observed across all groups. GPx, CAT, GST, and GRD levels decreased significantly after thawing, even in the L-serine-treated groups. Similar patterns reported in boar and human sperm studies [44,45,46] have been attributed to oxidative damage to enzymes, disruption of cellular redox cycling, and leakage of intracellular contents caused by membrane rupture.

Notably, increasing L-serine concentrations beyond 1 mM did not enhance protective effects and, in some cases, worsened outcomes. Higher doses (3–7 mM) were associated with elevated MDA levels and reduced enzyme activities, likely due to osmotic or redox imbalance under cryogenic conditions [47].

Overall, while supplementation with 1 mM L-serine enhanced antioxidant defense and structural preservation, post-thaw sperm quality remained lower than pre-freeze values across all groups. This underscores the inherent limitations of antioxidant supplementation alone. Future strategies should combine precisely dosed antioxidants like L-serine with optimized cryoprotectant formulations and cooling protocols to mitigate cryo-induced cellular stress further.

### 4.5. The Antioxidant Paradox and Dose-Dependent Detrimental Effect Across Preservation Methods

The results from both chilled and cryopreserved semen models highlight a critical and consistent pattern: antioxidant supplementation exhibits a biphasic dose response, in which moderate levels are beneficial, whereas excessive concentrations are detrimental. In the present study, low to moderate doses of L-serine (1–3 mM) significantly enhanced sperm function and antioxidant defenses, while higher levels (5–7 mM) impaired motility, viability, and mitochondrial integrity across both preservation methods.

This phenomenon reflects the well-documented antioxidant paradox. While moderate suppression of ROS enhances sperm preservation, excessive elimination of ROS may disrupt essential redox signaling required for capacitation and acrosome reaction [48]. Moreover, supra-physiological doses of L-serine may disturb cellular homeostasis through osmotic imbalance or mitochondrial redox disruption [49,50].

Notably, comparable dose-dependent declines in sperm quality have been observed with other antioxidants such as cysteine and resveratrol [51,52], reinforcing the need for precision dosing in semen extender formulations. Rather than adopting a “more-is-better” approach, antioxidant strategies must be carefully tailored to achieve a balance between providing protection and preserving physiological redox dynamics.

### 4.6. Chilled and Cryopreserved Semen: Differential Dose Responses

Although both preservation methods triggered oxidative stress, the optimal L-serine concentration differed between chilled and cryopreserved semen. During chilled storage at 17 °C, 3 mM L-serine showed the most favorable effects of protection by reducing lipid peroxidation and maintaining sperm motility, viability, and mitochondrial function during the first 2–3 days.

By contrast, during cryopreservation, sperm are subjected to a pre-freeze cooling phase at 5 °C, where cold shock induces membrane phase transitions, destabilization of lipid rafts, and disruption of mitochondrial activity. These changes are compounded by osmotic imbalance and dehydration associated with cryoprotectant exposure. Under these conditions, higher concentrations of L-serine (≥3 mM) failed to protect sperm and even worsened post-thaw outcomes. Instead, 1 mM supplementation was most effective in maintaining antioxidant enzyme activity, reducing lipid peroxidation, and preserving structural integrity after thawing.

This divergence underscores the distinct physiological challenges of each preservation stage. Chilled storage is characterized by gradual oxidative stress, which can be mitigated through enhanced glutathione metabolism, whereas cryopreservation involves acute cold-shock and osmotic trauma that demand a more delicate antioxidant balance. In this context, excessive L-serine may increase osmotic load or disrupt redox signaling, thereby compromising cryosurvival.

Taken together, these findings emphasize that antioxidant supplementation must be tailored to the preservation stage. L-serine at 3 mM is most suitable for mitigating oxidative damage during short-term chilled storage, whereas 1 mM is optimal during for cryopreservation, particularly across the vulnerable 5 °C cooling step. Such stage-specific dose optimization is essential for maximizing the protective benefits of antioxidants in boar semen preservation.

## 5. Conclusions

This study demonstrated that L-serine supplementation improved boar semen quality under both chilled and cryopreserved conditions, with clearly defined, dose-specific effects. At 3 mM, L-serine most effectively preserved motility, viability, and structural integrity during chilled storage, whereas 1 mM was more effective for cryopreservation, offering significant redox protection during the freeze–thaw process without inducing osmotic or oxidative imbalance. In contrast, higher concentrations (5–7 mM) were consistently detrimental to sperm quality across both storage methods.

These findings underscore the importance of dose-dependent and method-specific antioxidant strategies in the design of semen extenders. Tailoring antioxidant supplementation to the distinct physiological stressors of each preservation stage is crucial. Notably, L-serine represents a promising and cost-effective additive that can be precisely integrated into AI extender formulations to enhance semen quality, prolong storage life, and improve the reliability of artificial insemination systems in swine production.

However, it is important to acknowledge that this study was limited to in vitro semen quality assessments. No artificial insemination trials were conducted to directly evaluate fertility outcomes. Therefore, future in vivo studies are warranted to confirm whether the observed improvements in sperm quality translate into enhanced conception rates and litter performance under field conditions.

## Figures and Tables

**Table 1:** Effect of L-serine supplementation during chilled storage at 17 °C on boar semen quality over 1, 3, and 5 days.

Parameters	Control	L-Serine				SEM	*p*-Value
		1 mM	3 mM	5 mM	7 mM		
**Progressive motility (%)**							
Day 1	72.02 ^b^	74.60 ^b^	80.10 ^a^	75.10 ^b^	74.58 ^b^	1.12	0.0133
Day 3	63.23 ^c^	68.84 ^b^	74.47 ^a^	65.37 ^bc^	61.99 ^c^	1.30	<0.0001
Day 5	59.03 ^b^	61.40 ^b^	70.93 ^a^	57.88 ^b^	57.73 ^b^	1.31	<0.0001
**Viability (%)**							
Day 1	76.26 ^b^	76.63 ^b^	82.87 ^a^	74.57 ^bc^	70.90 ^c^	1.11	<0.0001
Day 3	68.41 ^b^	67.45 ^b^	76.47 ^a^	62.92 ^bc^	58.12 ^c^	1.46	<0.0001
Day 5	60.24 ^b^	63.16 ^b^	70.12 ^a^	57.98 ^bc^	53.77 ^c^	1.64	<0.0001
**Mitochondria function (%)**							
Day 1	69.59 ^b^	70.08 ^b^	79.07 ^a^	69.28 ^b^	69.66 ^b^	1.12	<0.0001
Day 3	62.32 ^b^	62.77 ^ab^	69.74 ^a^	57.54 ^bc^	54.95 ^c^	1.20	<0.0001
Day 5	49.07 ^bc^	52.69 ^b^	59.94 ^a^	49.12 ^bc^	43.87 ^c^	1.22	<0.0001
**Acrosome integrity (%)**							
Day 1	62.95 ^b^	63.81 ^b^	69.53 ^a^	60.63 ^b^	59.74 ^b^	0.88	<0.0001
Day 3	54.25 ^b^	57.90 ^b^	63.39 ^a^	56.57 ^b^	47.12 ^c^	1.13	<0.0001
Day 5	43.13 ^bc^	46.44 ^b^	53.05 ^a^	38.98 ^c^	32.59 ^d^	1.32	<0.0001

Values are presented as the mean ± SEM. Values within the same row with different superscripts (a–d) differ significantly (*p* < 0.05). Superscripts indicate comparisons between the L-serine concentrations on the same storage day.

**Table 2:** Effects of L-serine supplementation on lipid peroxidation and antioxidant enzyme activities during chilled storage of boar semen at 17 °C.

Treatments	Control	L-Serine				SEM	*p*-Value
		1 mM	3 mM	5 mM	7 mM		
**MDA (µmol/mL)**							
Day 1	0.67 ^bc^	0.65 ^ab^	0.56 ^a^	0.75 ^bc^	0.76 ^c^	0.02	<0.0001
Day 3	0.95 ^ab^	0.99 ^ab^	0.85 ^a^	1.04 ^b^	1.08 ^b^	0.03	0.0007
Day 5	1.10 ^b^	1.00 ^ab^	0.94 ^a^	1.00 ^ab^	1.13 ^b^	0.04	0.0070
**T-AOC (%)**							
Day 1	70.68 ^b^	71.67 ^ab^	72.07 ^ab^	71.50 ^ab^	72.24 ^b^	0.55	0.0500
Day 3	70.18 ^b^	71.97 ^ab^	72.55 ^a^	72.40 ^ab^	71.79 ^ab^	0.56	0.0376
Day 5	69.64 ^b^	70.83 ^ab^	72.05 ^a^	71.70 ^ab^	71.48 ^ab^	0.47	0.0249
**Gpx (U/mL)**							
Day 1	10.26 ^b^	11.65 ^ab^	12.87 ^a^	12.11 ^ab^	11.25 ^ab^	0.30	0.0422
Day 3	9.92 ^b^	10.53 ^ab^	11.73 ^a^	11.69 ^a^	11.73 ^a^	0.37	0.0024
Day 5	7.78 ^b^	11.05 ^ab^	12.31 ^a^	11.97 ^ab^	11.84 ^ab^	0.54	0.0320
**SOD (U/mL)**							
Day 1	813.33 ^b^	937.50 ^b^	918.33 ^b^	916.67 ^b^	1500.00 ^a^	73.87	0.0126
Day 3	875.00 ^b^	937.50 ^b^	875.00 ^b^	843.75 ^b^	1500.00 ^a^	66.77	0.0005
Day 5	791.67 ^b^	937.50 ^b^	906.25 ^b^	875.00 ^b^	1250.00 ^a^	43.17	0.0003
**CAT (U/mL)**							
Day 1	4.75 ^b^	6.67 ^ab^	9.83 ^a^	8.33 ^a^	2.75 ^b^	0.77	0.0189
Day 3	3.06 ^b^	3.56 ^ab^	7.56 ^a^	6.81 ^ab^	4.81 ^ab^	0.54	0.0246
Day 5	3.25 ^b^	4.06 ^ab^	7.50 ^a^	6.25 ^ab^	3.12 ^b^	0.55	0.0118
**GST (U/mL)**							
Day 1	25.71	24.73	25.95	21.78	26.54	0.59	0.0694
Day 3	24.80	23.81	25.75	26.42	25.63	0.51	0.6413
Day 5	25.29	24.38	23.45	25.87	23.95	0.68	0.8478
**GRD (U/mL)**							
Day 1	1.11	1.10	1.36	1.25	1.32	0.04	0.2875
Day 3	1.14	1.11	1.28	1.23	1.26	0.03	0.3153
Day 5	1.20	1.14	1.32	1.31	1.24	0.04	0.4975

Values within the same row with different superscript letters (a–c) are significantly different between L-serine concentrations on the same day (*p* < 0.05).

## Data Availability

The data are available upon request from the corresponding author.

## References

[1] Chanapiwat P., Kaeoket K. (2020). Cryopreservation of boar semen: Where we are. Thai J. Vet. Med..

[2] Bolarin A., Berndtson J., Tejerina F., Cobos S., Pomarino C., D’Alessio F., Blackburn H., Kaeoket K. (2024). Boar semen cryopreservation: State of the art, and international trade vision. Anim. Reprod. Sci..

[3] Peña F.J., O’Flaherty C., Ortiz Rodríguez J.M., Martín Cano F.E., Gaitskell-Phillips G.L., Gil M.C., Ortega Ferrusola C. (2019). Redox Regulation and Oxidative Stress: The Particular Case of the Stallion Spermatozoa. Antioxidants.

[4] Yeste M., Rodríguez-Gil J.E., Bonet S. (2017). Artificial insemination with frozen-thawed boar sperm. Mol. Reprod. Dev..

[5] Wang Y., Fu X., Li H. (2025). Mechanisms of oxidative stress-induced sperm dysfunction. Front. Endocrinol..

[6] Buhr M.M., Curtis E.F., Kakuda N.S. (1994). Composition and behavior of head membrane lipids of fresh and cryopreserved boar sperm. Cryobiology.

[7] Shabani Nashtaei M., Amidi F., Sedighi Gilani M.A., Aleyasin A., Bakhshalizadeh S., Naji M., Nekoonam S. (2017). Protective features of resveratrol on human spermatozoa cryopreservation may be mediated through 5′ AMP-activated protein kinase activation. Andrology.

[8] Sim W.C., Yin H.Q., Choi H.S., Choi Y.K., Kim S.K., Lee B.H. (2015). L-Serine Supplementation Attenuates Alcoholic Fatty Liver by Enhancing Homocysteine Metabolism in Mice and Rats. J. Nutr..

[9] Holecek M. (2022). Serine Metabolism in Health and Disease and as a Conditionally Essential Amino Acid. Nutrients.

[10] Rabattoni V., Marchesani F., Murtas G., Sacchi S., Mozzarelli A., Bruno S., Peracchi A., Pollegioni L., Campanini B. (2023). The human phosphorylated pathway: A multienzyme metabolic assembly for L-serine biosynthesis. FEBS J..

[11] Thananurak P., Chuaychu-noo N., Thélie A., Vongpralub T., Blesbois E. (2020). Different concentrations of cysteamine, ergothioneine, and serine modulate quality and fertilizing ability of cryopreserved chicken sperm. Poult. Sci..

[12] Kheawkanha T., Chankitisakul V., Thananurak P., Pimprasert M., Boonkum W., Vongpralub T. (2023). Solid storage supplemented with serine of rooster semen enhances higher sperm quality and fertility potential during storage at 5 °C for up to 120 h. Poult. Sci..

[13] Kong Y., He M., Gao J., Xu J., Lu N., Wu C., Sun L., Dai J. (2025). Comparative Analysis of Classic Semen Extenders for Frozen–Thawed Boar Semen. Animals.

[14] de Oliveira M.B., Ferreira H.N. (2019). Importância da adição de antioxidantes ao sêmen criopreservado de carneiros. Multidiscip. Rev..

[15] Khoi H.X., Shimizu K., Yoneda Y., Minagawa I., Abe Y., Kuwabara Y., Sasanami T., Kohsaka T. (2021). Monitoring the reactive oxygen species in spermatozoa during liquid storage of boar semen and its correlation with sperm motility, free thiol content and seasonality. Andrologia.

[16] Hallberg I., Morrell J.M. (2024). Sperm quality and in vitro fertilizing ability of boar spermatozoa stored at 4 °C versus conventional storage for 1 week. Front. Vet. Sci..

[17] Chankitisakul V., Boonkum W., Kaewkanha T., Pimprasert M., Ratchamak R., Authaida S., Thananurak P. (2022). Fertilizing ability and survivability of rooster sperm diluted with a novel semen extender supplemented with serine for practical use on small holder farms. Poult. Sci..

[18] Ratchamak R., Authaida S., Koedkanmark T., Boonkum W., Semaming Y., Chankitisakul V. (2024). Dietary supplementation with ginseng extract enhances testicular function, semen preservation, and fertility rate of mature and aging Thai native roosters. Theriogenology.

[19] Vongpralub T., Thananurak P., Sittikasamkit C., Chuawongboon P., Duangjinda M., Boonkum W., Chankitisakul V. (2018). Comparison of Effects of Different Antioxidants Supplemented to Long-term Extender on Boar Semen Quality Following Storage at 17 °C. Thai J. Vet. Med..

[20] Ratchamak R., Vongpralub T., Boonkum W., Chankitisakul V. (2019). Cryopreservation and quality assessment of boar semen collected from bulk samples. Vet. Med..

[21] Ratchamak R., Ratsiri T., Kheawkanha T., Vongpralub T., Boonkum W., Chankitisakul V. (2020). Evaluation of cryopreserved boar semen after supplementation sericin form silkworm (*Bombyx mori*) in semen extender. Anim. Sci. J..

[22] Karirat T., Saengha W., Deeseenthum S., Ma N.L., Sutthi N., Wangkahart E., Luang-In V. (2023). Data on exopolysaccharides produced by *Bacillus* spp. from cassava pulp with antioxidant and antimicrobial properties. Data Brief.

[23] Fontagné-Dicharry S., Larroquet L., Dias K., Cluzeaud M., Heraud C., Corlay D. (2018). Effects of dietary oxidized fish oil supplementation on oxidative stress and antioxidant defense system in juvenile rainbow trout (*Oncorhynchus mykiss*). Fish Shellfish Immunol..

[24] Misra H.P., Fridovich I. (1972). The role of superoxide anion in the autoxidation of epinephrine and a simple assay for superoxide dismutase. J. Biol. Chem..

[25] Weydert C.J., Cullen J.J. (2010). Measurement of superoxide dismutase, catalase and glutathione peroxidase in cultured cells and tissue. Nat. Protoc..

[26] Zablotowicz R.M., Hoagland R.E., Locke M.A., Hickey W.J. (1995). Glutathione-S transferase activity and metabolism of glutathione conjugates by rhizosphere bacteria. Appl. Environ. Microbiol..

[27] Hammerstedt R.H., Graham J.K., Nolan J.P. (1990). Cryopreservation of mammalian sperm: What we ask them to survive. J. Androl..

[28] Watson P.F. (2000). The causes of reduced fertility with cryopreserved semen. Anim. Reprod. Sci..

[29] Maralani M.N., Movahedian A., Javanmard S.H. (2012). Antioxidant and cytoprotective effects of L-Serine on human endothelial cells. Res. Pharm. Sci..

[30] Zhou X., He L., Wu C., Zhang Y., Wu X., Yin Y. (2017). Serine alleviates oxidative stress via supporting glutathione synthesis and methionine cycle in mice. Mol. Nutr. Food Res..

[31] Amelio I., Cutruzzolá F., Antonov A., Agostini M., Melino G. (2014). Serine and glycine metabolism in cancer. Trends Biochem. Sci..

[32] Zhou X., Zhang Y., Wu X., Wan D., Yin Y. (2018). Effects of Dietary Serine Supplementation on Intestinal Integrity, Inflammation and Oxidative Status in Early-Weaned Piglets. Cell. Physiol. Biochem. Int. J. Exp. Cell. Physiol. Biochem. Pharmacol..

[33] Fan J., Ye J., Kamphorst J.J., Shlomi T., Thompson C.B., Rabinowitz J.D. (2014). Quantitative flux analysis reveals folate-dependent NADPH production. Nature.

[34] Cerolini S., Maldjian A., Pizzi F., Gliozzi T.M. (2001). Changes in sperm quality and lipid composition during cryopreservation of boar semen. Reproduction.

[35] Hu R., Yang X., Gong J., Lv J., Yuan X., Shi M., Fu C., Tan B., Fan Z., Chen L. (2024). Patterns of alteration in boar semen quality from 9 to 37 months old and improvement by protocatechuic acid. J. Anim. Sci. Biotechnol..

[36] Pollock K., Yu G., Moller-Trane R., Koran M., Dosa P.I., McKenna D.H., Hubel A. (2016). Combinations of Osmolytes, Including Monosaccharides, Disaccharides, and Sugar Alcohols Act in Concert During Cryopreservation to Improve Mesenchymal Stromal Cell Survival. Tissue Eng. Part C Methods.

[37] Menegat M.B., Mellagi A.P., Bortolin R.C., Menezes T.A., Vargas A.R., Bernardi M.L., Wentz I., Gelain D.P., Moreira J.C., Bortolozzo F.P. (2017). Sperm quality and oxidative status as affected by homogenization of liquid-stored boar semen diluted in short- and long-term extenders. Anim. Reprod. Sci..

[38] Li J., Zhao W., Zhu J., Ju H., Liang M., Wang S., Chen S., Ferreira-Dias G., Liu Z. (2023). Antioxidants and Oxidants in Boar Spermatozoa and Their Surrounding Environment Are Associated with AMPK Activation during Liquid Storage. Vet. Sci..

[39] Sakamoto T., Imai H. (2017). Hydrogen peroxide produced by superoxide dismutase SOD-2 activates sperm in Caenorhabditis elegans. J. Biol. Chem..

[40] Hussain S.P., Amstad P., He P., Robles A., Lupold S., Kaneko I., Ichimiya M., Sengupta S., Mechanic L., Okamura S. (2004). p53-induced up-regulation of MnSOD and GPx but not catalase increases oxidative stress and apoptosis. Cancer Res..

[41] Yuzhalin A.E., Kutikhin A.G. (2012). Inherited variations in the SOD and GPX gene families and cancer risk. Free Radic. Res..

[42] Njoroge W.E., Zhu Z., Umehara T., Yamanaka T., Zeng W., Okazaki T., Shimada M. (2025). Synthesis of functional enzymes involved in glutathione production during linear motility in boar sperm. Free Radic. Biol. Med..

[43] Yeste M., Flores E., Estrada E., Bonet S., Rigau T., Rodríguez-Gil J.E. (2013). Reduced glutathione and procaine hydrochloride protect the nucleoprotein structure of boar spermatozoa during freeze–thawing by stabilising disulfide bonds. Reprod. Fertil. Dev..

[44] Zhang B., Wang Y., Wu C., Qiu S., Chen X., Cai B., Xie H. (2021). Freeze-thawing impairs the motility, plasma membrane integrity and mitochondria function of boar spermatozoa through generating excessive ROS. BMC Vet. Res..

[45] Alvarez J.G., Storey B.T. (1992). Evidence for Increased Lipid Peroxidative Damage and Loss of Superoxide Dismutase Activity as a Mode of Sublethal Cryodamage to Human Sperm During Cryopreservation. J. Androl..

[46] Castellini C., Placidi M., Barbonetti A., Tatone C., Emidio G.D. (2024). Mechanisms underlying human sperm cryodamage: The role of reactive oxygen species (ROS) and antioxidants. RIVER J..

[47] Sadeghi N., Boissonneault G., Tavalaee M., Nasr-Esfahani M.H. (2023). Oxidative versus reductive stress: A delicate balance for sperm integrity. Syst. Biol. Reprod. Med..

[48] Tvrdá E., Mackovich A., Greifová H., Lukáč N. (2016). Lycopene offers protection against oxidative damage in frozen-thawed bovine semen. Sci. Pap. Anim. Sci. Biotechnol..

[49] He L., Ding Y., Zhou X., Li T., Yin Y. (2023). Serine signaling governs metabolic homeostasis and health. Trends Endocrinol. Metab..

[50] Engel A.L., Lorenz N.I., Klann K., Münch C., Depner C., Steinbach J.P., Ronellenfitsch M.W., Luger A.L. (2020). Serine-dependent redox homeostasis regulates glioblastoma cell survival. Br. J. Cancer.

[51] Funahashi H., Sano T. (2025). Select antioxidants improve the function of extended boar semen stored at 10 °C. Theriogenology.

[52] Kaeoket K., Chanapiwat P. (2023). The beneficial effect of resveratrol on the quality of frozen-thawed boar sperm. Animals.

